# Neuroinformatics and the The Insight ToolKit

**DOI:** 10.3389/fninf.2015.00005

**Published:** 2015-03-26

**Authors:** Brian B. Avants, Hans J. Johnson, Nicholas J. Tustison

**Affiliations:** ^1^Penn Image Computing and Science Laboratory, University of PennsylvaniaPhiladelphia, PA, USA; ^2^Department of Psychiatry, University of IowaIowa City, IA, USA; ^3^Department of Radiology and Medical Imaging, University of VirginiaCharlottesville, VA, USA

**Keywords:** open source, segmentation, registration, C++, ITK

Open source software is a fundamental mechanism for storing and disseminating knowledge. This role is critical to science and is arguably equally, if not more, important than traditional publication venues in terms of practical and long-term impact. Well-constructed software libraries are repositories of both practical and theoretical content that not only serve data analysis but also help educate current and future scientists which substantially augments traditional publication. Highly software-engineered resources such as BLAS, Armadillo, Eigen, SciPy, R and its packages, Theano and many more are critical to the science of the Large Hadron Collider, Human Genome Project, the Human Connectome Project, and to smaller projects conducted at Universities throughout the world.

The Insight ToolKit (ITK) is a computational library and repository of knowledge with a specific focus toward medical image processing. The primary goal of this article collection is to summarize the recent and varied contributions of the ITK community to neuroinformatics and related fields. Following several discussions during weekly teleconferences and at developer meetings, we made the call for contributions to this research topic on *Fri Mar 22 15:48:30 EDT 2013* in an email sent to the ITK discussion list. Initially, we anticipated approximately 10 submissions. By the close of submission (after one extension period), we received 17 excellent submissions that represent a large range of ITK functionality and application. The overall quality is a reflection of ITK's broad impact as well as the commitment of the community to reproducibility, openness, and high standards. Furthermore, thanks to cooperation from both authors and reviewers, we were able to receive, review, and publish these articles within a year.

ITK is intended to be an international, open resource that accelerates image-based science and enables advanced scientific computing across major platforms. ITK began in 1999 as a collaboration between academia and industry to enable robust processing of Visible Human data using open science tools and is supported by the National Library of Medicine under funding directed, historically, by Terry Yoo. Since that time, ITK has served as a core library supporting several well-known packages such as Elastix, ITK-SNAP, Advanced Normalization Tools, DTIStudio, BrainsSuite, Osirix, Slicer, and others. ITK is widely used in industry (oftentimes without acknowledgment) and has led to other large software projects unrelated to the medical imaging field such as the Orfeo Toolbox initiative sponsored by the French space agency (CNES). ITK's prevalence in academia is evidenced by the more than 1500 papers in Google Scholar that explicitly reference the ITK (http://scholar.google.com/scholar?q=ITK+%22Insight+ToolKit+%22), although we expect there are many more that use but do not reference ITK.

The widespread adoption of the ITK is due to a thorough and publicly visible testing framework, a consistent coding style, and dimensionality-free algorithm implementations that are flexible with respect to data type (scalar, vector, tensor, etc…). That is, one need only implement an algorithm once and it is functional in two to N-dimensions. This advantage is achieved via the underlying templated C++ code base. While this code may present a challenge to some, we have found that the abundance of examples within ITK form a set of natural stepping stones that, if followed, eventually allow one to become proficient in the use of ITK with the eventual possibility of becoming an ITK contributor. For instance, one might study the pipeline that adds two images and build knowledge from there. It is likely that nearly every paper contributed to this topic has at least one author that learned ITK coding in this way.

This research topic captures ITK's design benefits, its concomitant scope as well as its impact through derived applications. McCormick et al provide a good introduction to the philosophy, design and maintenance of ITK and its role in reproducible science (McCormick et al., 2014). ITK is also being used for production quality diffusion tensor processing (Ipek et al., 2014; Verde et al., 2014) and structural network analysis (Duda et al., 2014). ITK's potential for enabling rapid MRI-assisted clinical diagnosis (Denis et al., 2014) via GPU-accelerated image registration is highlighted in Shamonin et al. A pair of articles shows ITK's value in assisting surgical intervention (Drakopoulos et al., 2014; Liu et al., 2014). A central theme across several papers in this topic, including our own, is ITK's robust and scalable image processing strategies that are meeting the demands for curation and interpretation of “big data” in medical imaging (Wang and Yushkevich, 2013; Young and Johnson, 2013). Both the new registration framework (Fred et al., 2014) and level set framework (Mosaliganti et al., 2013) are introduced to the literature for the first time in this topic. Tustison et al also contributes a new variant of the SyN registration method that demonstrates performance improvements over the original algorithm (Tustison and Avants, 2013). Finally, one of the most important contributions, SimpleITK, is outlined by Lowekamp et al. (2013). SimpleITK is an exciting generalized interface that promises to increase the accessibility of ITK to scientists more familiar with scripting languages such as python, *R* or Matlab.

To our knowledge, this is the first collection of articles that focuses on the science and engineering of the ITK. Organizing and reviewing the articles submitted to this collection encouraged our belief that the software itself is instrumental to the scientific process and changes, fundamentally, the way work is performed—*for the better*. ITK's extensibility and customizability is critical to imaging science which is constantly facing new problem domains. Additionally, ITK's near limitless flexibility in combining tools and features enables new problems to be addressed, new scientific questions to be asked and new standards to be established. As an example, ITK was arguably instrumental, historically, in establishing the translational value of diffeomorphic image registration as a new baseline for registration quality. Several concerns are commonly raised regarding the toolkit: Is ITK just a software library or is it a core scientific tool? We argue that ITK is both: parts of the toolkit define canonical “library” algorithms and reference implementations whereas others encode frameworks and tools with possibilities yet to be realized. Thus, ITK is both an archive of established ideas and a living document of current ideas (e.g., the registration and level set frameworks) that is continually explored and extended by the community.

While ITK is intended to encourage best practices for research and reproducible science endeavors, our feeling is that much more work must be done to educate the scientific community about practices that will augment reproducibility by minimizing bugs, reducing software maintenance burden and increase the impact of a domain expert's software development efforts. To make this happen, we must translate not only the philosophy of open science but also its instantiations into the hands of students, post-docs, and even professors. It is an interesting irony that the software industry may currently be better, in many ways, at verification and openness than are many scientists. The current publishing practice where researchers self-report the performance of their proposed algorithm with possible juxtaposition with other relevant algorithms is inadequate as it is often fraught with problems. Much of the research literature contains little to no pseudo-code much less access to the actual algorithmic implementation and crucial parameters are often glossed over or omitted altogether [Kovacevic, J.: From the editor-in-chief. IEEE Trans Image Proc 15(12) (Dec 2006)]. Thus, the interested reader is often limited to the corresponding evaluations to assess performance. However, such evaluations are often corrupted by methodological flaws such as selection [http://www.ncbi.nlm.nih.gov/pmc/articles/PMC2841687/] and instrumentation http://www.ncbi.nlm.nih.gov/pmc/articles/PMC3766821/] biases. To improve uptake among scientists, we must establish that these open science practices save time, in the long run, by encouraging organization and discipline as well as by improving communication.

Inclusivity is also critical to ITK's future. While the ITK developer community has spent over 10 years establishing a quality core, this has led, of necessity, to a degree of exclusion: software contributions that do not meet the testing, implementation and style metrics established by ITK cannot be included in the toolkit. An alternative model is provided by Python wherein software can be developed and combined much more freely—with minimal to no code review. This latter approach, we feel, is too disorderly. However, ITK may be too restrictive. *R* provides another model: new contributions are built from established skeletons with very clearly defined program development, testing, and documentation procedures. The ITK community might seek to establish similar well-documented (and publicized, accessible) guidelines. The recent ITKv4 modularization, adoption of the Gerrit review system, and accommodating remote modules in external repositories, such as github, are moving ITK in this direction. This new technologically-enabled research model recalls the not-so-distant days of hand-written lab notebooks wherein researchers were encouraged to *keep every detail without deletion*. Flexible resources such as github and bitbucket may be modern incarnations of this very same idea although they are currently relatively unused by scientists working outside of computation. The ITK community should also continue to take very seriously the need to integrate with the “script based science” of python, *R*, matlab, Julia, etc. SimpleITK is again a step in the right direction but more contributors and users are essential to the building up of this important branch of the ITK tree. We believe that aspects of all of the above are necessary to build community bidirectionally and ensure longevity and increase the impact of the ITK.

Open source projects, in general, struggle to gain recognition as scientifically valuable within traditional academic venues. In part, because these successes are often undocumented, it remains uncertain how academics might expect to get credit from their institutions for contributing to open source software. There is currently no straightforward answer to this although, again, history shows that well-designed, well-implemented numerical, and scientific software is an investment that yields returns far beyond what might be expected, per dollar, in many other domains. While it is well-known in industry and even provincially (thanks to well-known disruptions of productivity that occur in large-scale use of operating systems developed by major software companies) that good software is challenging to create, there remains an attitude exemplified by the less-knowledgable inquiry “don't you just run some program?” when it comes to performing image-based science.

Several important aspects of ITK are not covered in topic articles. These include recent advances in SimpleITK which is under rapid development. There is also a detailed and updated software guide being written under the aegis of the Insight Consortium. An online set of ITK examples is being contributed by Kitware. Both of these are constantly growing in depth and scope. We refer readers to the ITK website and discussion boards for more up to date details on these topics. ITK has also, historically, avoided functional MRI processing and statistical issues and this collection reflects that. While substantial effort has been made in ITK's progeny [e.g., ANTsR (http://stnava.github.io/ANTsR/)], many users are unaware of these resources or still lack the necessary technical skills to install and employ source that, while relatively mature for research grade code, remains “work in progress.” Hopefully, this will change in the future.

We would like to close this introduction by thanking Terry Yoo and the National Library of Medicine for funding this work and supporting this research topic. The Insight Consortium, with major contributions from its president, Hans J. Johnson, has also been instrumental to extending the vision and quality established by the original ITK developers. The toolkit would not continue to exist without the ITK user community and thus the community deserves perhaps the greatest thanks of all. Finally, we must thank both the authors and reviewers of the submitted articles, and the frontiers organization, for responding in an organized and timely fashion to our editorial demands.

## Conflict of interest statement

The authors declare that the research was conducted in the absence of any commercial or financial relationships that could be construed as a potential conflict of interest.

## References

[B1] Denis P. S. Esther E. B. Boudewijn P. F. L. Marion S. Stefan K. Marius S.. (2014). Fast parallel image registration on CPU and GPU for diagnostic classification of Alzheimer's disease. Front. Neuroinform. 7:50. 10.3389/fninf.2013.00050 24474917 PMC3893567

[B2] Drakopoulos F. Foteinos P. Liu Y. Chrisochoides N. P. (2014). Toward a real time multi-tissue Adaptive Physics-Based Non-Rigid Registration framework for brain tumor resection. Front. Neuroinform. 8:11. 10.3389/fninf.2014.00011 24596553 PMC3925835

[B3] Duda J. T. Cook P. A. Gee J. C. (2014). Reproducibility of graph metrics of human brain structural networks. Front. Neuroinform. 8:46. 10.3389/fninf.2014.00046 24847245 PMC4019854

[B4] Fred W. M. Nora P. Matthias H. Luzia G. (2014). Spatial cognition, body representation and affective processes: the role of vestibular information beyond ocular reflexes and control of posture. Front. Integr. Neurosci. 8:44. 10.3389/fnint.2014.00044 24904327 PMC4035009

[B5] Ipek O. Mahshid F. Joy M. Francois B. Zhexing L. Guido G.. (2014). DTIPrep: quality control of diffusion-weighted images. Front. Neuroinform. 8:4. 10.3389/fninf.2014.00004 24523693 PMC3906573

[B6] Liu Y. Kot A. Drakopoulos F. Yao C. Fedorov A. Enquobahrie A.. (2014). An ITK implementation of a physics-based non-rigid registration method for brain deformation in image-guided neurosurgery. Front. Neuroinform. 8:33. 10.3389/fninf.2014.00033 24778613 PMC3985035

[B7] Lowekamp B. C. Chen D. T. Ibáñez L. Blezek D. (2013). The design of SimpleITK. Front. Neuroinform. 7:45. 10.3389/fninf.2013.00045 24416015 PMC3874546

[B8] McCormick M. Liu X. Jomier J. Marion C. Ibanez L. (2014). ITK: enabling reproducible research and open science. Front. Neuroinform. 8:13. 10.3389/fninf.2014.00013 24600387 PMC3929840

[B9] Mosaliganti K. R. Gelas A. Megason S. G. (2013). An efficient, scalable, and adaptable framework for solving generic systems of level-set PDEs. Front. Neuroinform. 7:35. 10.3389/fninf.2013.00035 24501592 PMC3872740

[B10] Tustison N. J. Avants B. B. (2013). Explicit B-spline regularization in diffeomorphic image registration. Front. Neuroinform. 7:39. 10.3389/fninf.2013.00039 24409140 PMC3870320

[B11] Verde A. R. Budin F. Berger J. B. Gupta A. Farzinfar M. Kaiser A.. (2014). UNC-Utah NA-MIC framework for DTI fiber tract analysis. Front. Neuroinform. 7:51. 10.3389/fninf.2013.00051 24409141 PMC3885811

[B12] Wang H. Yushkevich P. A. (2013). Multi-atlas segmentation with joint label fusion and corrective learning-an open source implementation. Front. Neuroinform. 7:27. 10.3389/fninf.2013.00027 24319427 PMC3837555

[B13] Young K. E. Johnson H. J. (2013). Robust multi-site MR data processing: iterative optimization of bias correction, tissue classification, and registration. Front. Neuroinform. 7:29. 10.3389/fninf.2013.00029 24302911 PMC3831347

